# Multicenter Validation of a Unified Evidence-Based Treatment Protocol Focusing on Clazosentan for Managing Subarachnoid Hemorrhage

**DOI:** 10.3390/jcm14103423

**Published:** 2025-05-14

**Authors:** Hiroshi Kondo, Daizo Ishii, Masashi Kuwabara, Takeshi Hara, Kaoru Kurisu, Masayuki Sumida, Fusao Ikawa, Shinji Ohba, Atsushi Tominaga, Naohiko Obayashi, Kazuhiko Kuroki, Takashi Sadatomo, Osamu Hamasaki, Shigeyuki Sakamoto, Toshinori Matsushige, Yosuke Watanabe, Hayato Araki, Masaru Abiko, Nobuhiko Ichinose, Atsumi Takenobu, Nobutaka Horie

**Affiliations:** 1 Department of Neurosurgery, Graduate School of Biomedical and Health Sciences, Hiroshima University, Hiroshima 734-8551, Japan; daiishii@hiroshima-u.ac.jp (D.I.); m214028@gmail.com (M.K.); thara@hiroshima-u.ac.jp (T.H.); horie@hiroshima-u.ac.jp (N.H.); 2 Department of Neurosurgery, National Hospital Organization Kure Medical Center and Chugoku Cancer Center, Kure 737-0023, Japan; ooba.shinji.wj@mail.hosp.go.jp; 3 Chugoku Rosai Hospital, Kure 737-0193, Japan; kuka422@chugokuh.johas.go.jp; 4 Department of Neurosurgery, Hiroshima Red Cross Hospital and Atomic-bomb Survivors Hospital, Hiroshima 730-8619, Japan; sumidamasa@gmail.com; 5 Department of Neurosurgery, Shimane Prefectural Central Hospital, Izumo 693-8555, Japan; fikawa@hiroshima-u.ac.jp; 6 Department of Neurosurgery and Neuroendovascular Therapy, Hiroshima Prefectural Hospital, Hiroshima 734-8530, Japan; atomin1963@gmail.com; 7 Department of Neurosurgery, Matsue Red Cross Hospital, Matsue 690-8506, Japan; n972061@yahoo.co.jp; 8 Department of Neurosurgery, JA Hiroshima General Hospital, Hatsukaichi 738-8503, Japan; kkurocky6576@yahoo.co.jp; 9 Department of Neurosurgery, National Hospital Organization Higashihiroshima Medical Center, Higashihiroshima 739-0041, Japan; tasanetomo@gmail.com; 10 Department of Neurosurgery, Miyoshi Central Hospital, Miyoshi 728-8502, Japan; o.hamasaki7745@city.miyoshi.hiroshima.jp; 11 Department of Neurosurgery, Itsukaichi Memorial Hospital, Itsukaichi 731-5156, Japan; shigeyukisakamoto@gmail.com; 12 Department of Neurosurgery and Interventional Neuroradiology, Hiroshima City North Medical Center Asa Citizens Hospital, Hiroshima 731-0293, Japan; teruteru728@yahoo.co.jp; 13 Department of Neurosurgery, Matsuyama Red Cross Hospital, Matsuyama 790-8524, Japan; watanabeyosuke2001@yahoo.co.jp; 14 Department of Neurosurgery, Araki Neurosurgical Hospital, Hiroshima 733-0821, Japan; haraki@arakihp.jp; 15 Department of Neurosurgery, JA Onomichi General Hospital, Onomichi 722-8508, Japan; tenmab_s@yahoo.co.jp; 16 Department of Neurosurgery, Ichinose Hospital, Hiroshima 730-0042, Japan; 17 Department of Neurosurgery, Teraoka Memorial Hospital, Fukuyama 729-3103, Japan; neurostake@gmail.com

**Keywords:** clazosentan, aneurysmal subarachnoid hemorrhage, unified treatment protocol, vasospasm, systemic complications

## Abstract

**Background/Objectives**: Effective management of aneurysmal subarachnoid hemorrhage (aSAH) requires an evidence-based treatment protocol. This study examines the outcomes of a unified, multicenter protocol emphasizing postoperative clazosentan as the first-line treatment for vasospasm. **Methods**: A standardized protocol prioritizing systemic management with clazosentan for vasospasm was implemented in April 2023. Cases treated between April 2022 and March 2024 were categorized into four groups: preprotocol fasudil treatment (PrF), preprotocol clazosentan treatment (PrC), postprotocol fasudil treatment (PoF), and postprotocol clazosentan treatment (PoC); these groups were analyzed. **Results**: Among 407 registered cases, 322 were eligible for analysis (PrF, 128; PrC, 69; PoF, 28; PoC, 97). PoC exhibited significantly lower angiographic vasospasm rates and had a lower incidence of symptomatic vasospasm compared with PrF (*p* = 0.048, *p* = 0.057). Logistic regression identified the clazosentan protocol as a predictive factor for vasospasm reduction (*p* = 0.02, OR 0.46 [0.22–0.94]; *p* = 0.022, OR 0.38 [0.16–0.91]). PoC experienced less fluid retention than the PrC (*p* < 0.001). Logistic regression confirmed protocol adherence with protocol reduced complications (*p* < 0.001, OR 0.24 [0.11–0.52]), included fluid retention (*p* < 0.001, OR 0.088 [0.03–0.29]). In older patients, no significant differences in vasospasm or complications were observed between PrF and PoC, but a trend toward reduced complications was observed in World Federation of Neurosurgical Societies (WFNS) grade V cases. **Conclusions**: Clazosentan-first protocol effectively reduces vasospasm and complications in aSAH management. It is also safe for older patients and those with WFNS grade V, offering a promising treatment strategy.

## 1. Introduction

The management of subarachnoid hemorrhage caused by ruptured cerebral aneurysm (aSAH) involves integrating various factors, such as surgical procedures, postoperative care—including fluid management and medication—and other treatments, tailored to the patient’s age, disease severity, aneurysm morphology and location, and underlying conditions. However, in practice, these decisions are often guided by the expertise and experience of the facility or attending physician, resulting in a lack of standardization in treatment and management. In Japan, a phase III study demonstrated the efficacy of clazosentan in preventing postoperative cerebral vasospasm compared to placebo [1]. Additionally, several clinical practice reports have been published [2,3]. While these developments have expanded the options for aSAH management, there remains a need for evidence-based standardized protocols. At the same time, treatment decisions must consider the individual patient’s condition in clinical practice.

This report presents the outcomes of an aSAH management protocol developed at Hiroshima University Hospital and its affiliated institutions, applied in real-world clinical settings.

## 2. Materials and Methods

The “Hiroshima University Version of the Subarachnoid Hemorrhage Management Protocol” was developed through collaboration between Hiroshima University and its affiliated hospitals. This protocol outlines procedures for initial response and examinations, surgical intervention, spinal fluid management, postoperative systemic care—including fluid balance—management of delayed cerebral ischemia (DCI), with a focus on clazosentan as the primary treatment, response to DCI onset, postoperative imaging schedules, nutrition, and rehabilitation. Key aspects of the protocol are summarized in Table 1. A central feature of the protocol is the use of clazosentan as the first-line prophylactic treatment for DCI. Additionally, to mitigate fluid retention, a known side effect of clazosentan, patients are managed with normovolemia by regulating infusion volumes while avoiding dehydration. Based on this protocol, each patient’s condition was assessed by the respective facility and attending neurosurgeons, who exercised discretion in determining the appropriate treatment.

Patients with aSAH treated at Hiroshima University Hospital and its affiliated hospitals between April 2022 and March 2024 were included in this study. While the protocol was officially introduced in April 2023, its implementation varied across facilities due to logistical adjustments, resulting in a transition period. Notably, clazosentan was frequently used even before the protocol’s formal adoption. And as a general principle, clazosentan monotherapy is the first-line choice for DCI prophylaxis after the introduction of the protocol. However, the final decision regarding whether to use clazosentan or fasudil is left to the discretion of each physician or institution based on their clinical judgment. Therefore, patients were categorized into four groups based on the timeline relative to the protocol introduction and the medication used for DCI management (fasudil or clazosentan): preprotocol fasudil treatment (PrF) group, preprotocol clazosentan treatment (PrC) group, postprotocol fasudil treatment (PoF) group, and postprotocol clazosentan treatment (PoC) group. Data analysis included patient demographics (age, sex, medical history, World Federation of Neurosurgical Societies [WFNS] grade, Fisher group), surgical treatment type (craniotomy or endovascular surgery), postoperative DCI management (medications used), radiographic cerebral vasospasm (defined as ≥50% stenosis), symptomatic vasospasm, complications (systemic complications defined as any adverse events; fluid retention defined as pulmonary edema, pulmonary effusion or congestive heart failure), and modified Rankin Scale (mRS) scores at discharge.

This multicenter collaborative study was approved by the Ethics Committee of Hiroshima University through a single review process (Approval No. E2023-0247). Informed consent was obtained through an opt-out process at the participating institutions.

### Statistical Analysis

Continuous variables were reported as means, and categorical variables were presented as counts (percentages). The Mann–Whitney U test was used to analyze continuous variables, while Fisher’s exact test was applied for categorical variables. Logistic regression analysis was conducted to evaluate angiographic vasospasm, symptomatic vasospasm, systemic complications, and fluid retention, considering age (using the mean as a cutoff), sex, WFNS grade, surgical procedure, and treatment group as potential confounders. A *p*-value of <0.05 was considered statistically significant. All statistical analyses were performed using JMP Pro 18.

## 3. Results

**Figure 1:**
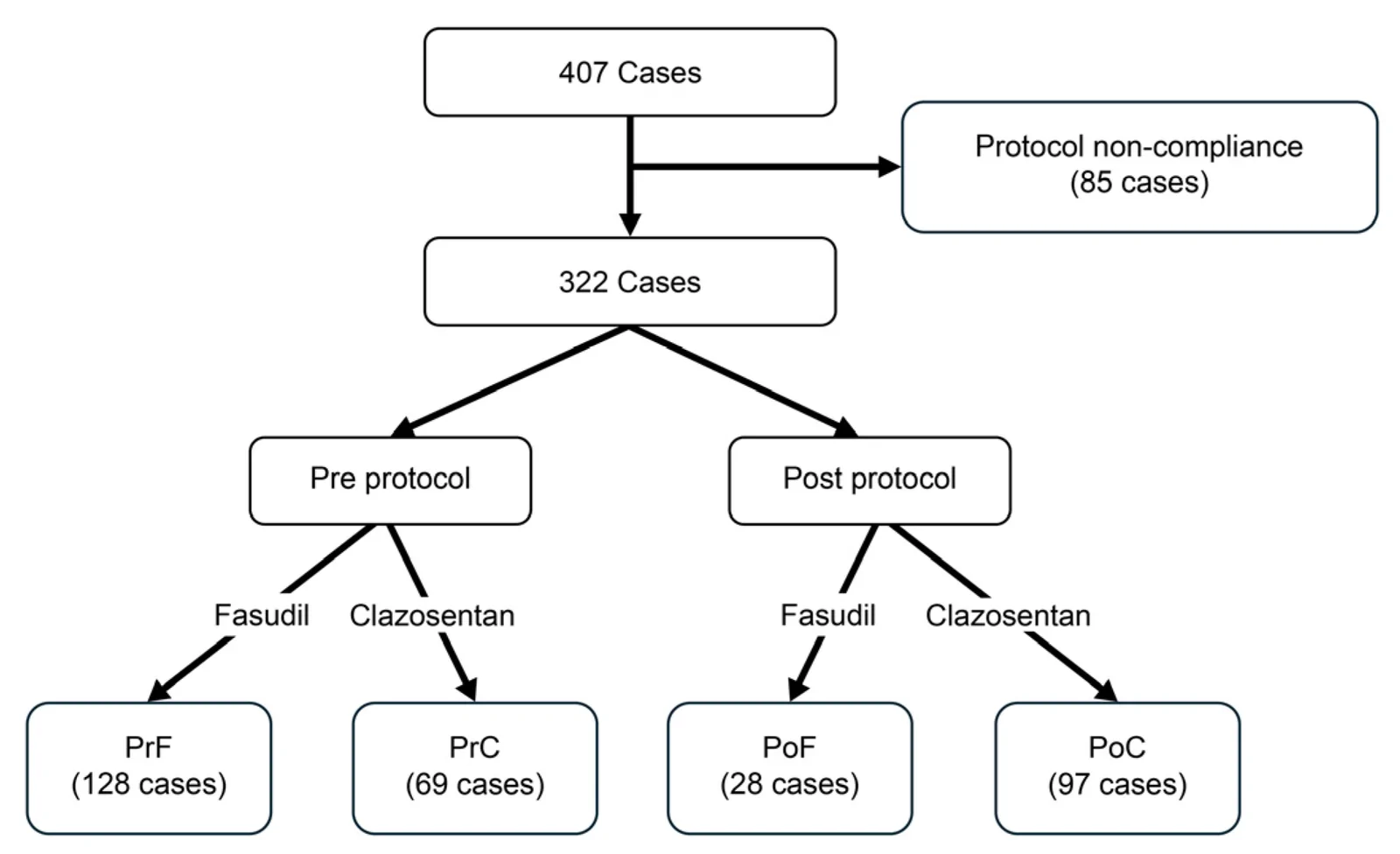
Flowchart of study inclusion and exclusion criteria and participant group allocation into four categories.

During the study period, 407 cases were registered from the affiliated facilities. Of these, 79 cases were excluded due to the absence of DCI treatment, including those with the most severe aSAH conditions. Additionally, six cases were excluded for not adhering to the protocol for DCI management, such as the combined use of clazosentan and fasudil, resulting in a total of 85 exclusions. Consequently, 322 cases were included in the final analysis and categorized as follows: PrF group (128 cases), PrC group (69 cases), PoF group (28 cases), and PoC group (97 cases) (Figure 1). During treatment, three patients in the PrC group and six in the PoC group were switched from clazosentan to fasudil. Among the cases, fluid retention was the underlying cause in two cases in the PrC and PoC groups. Regarding the distribution of systemic complications, there were 39 cases related to fluid retention (including pulmonary edema, pulmonary effusion, and heart failure), 28 cases of pneumonia, 15 cases of other infections, 18 cases of electrolyte abnormalities, 4 cases of deep vein thrombosis, and 23 cases of other complications (with overlap).

### 3.1. Comparison Between PrF and PoC

**Figure 2:**
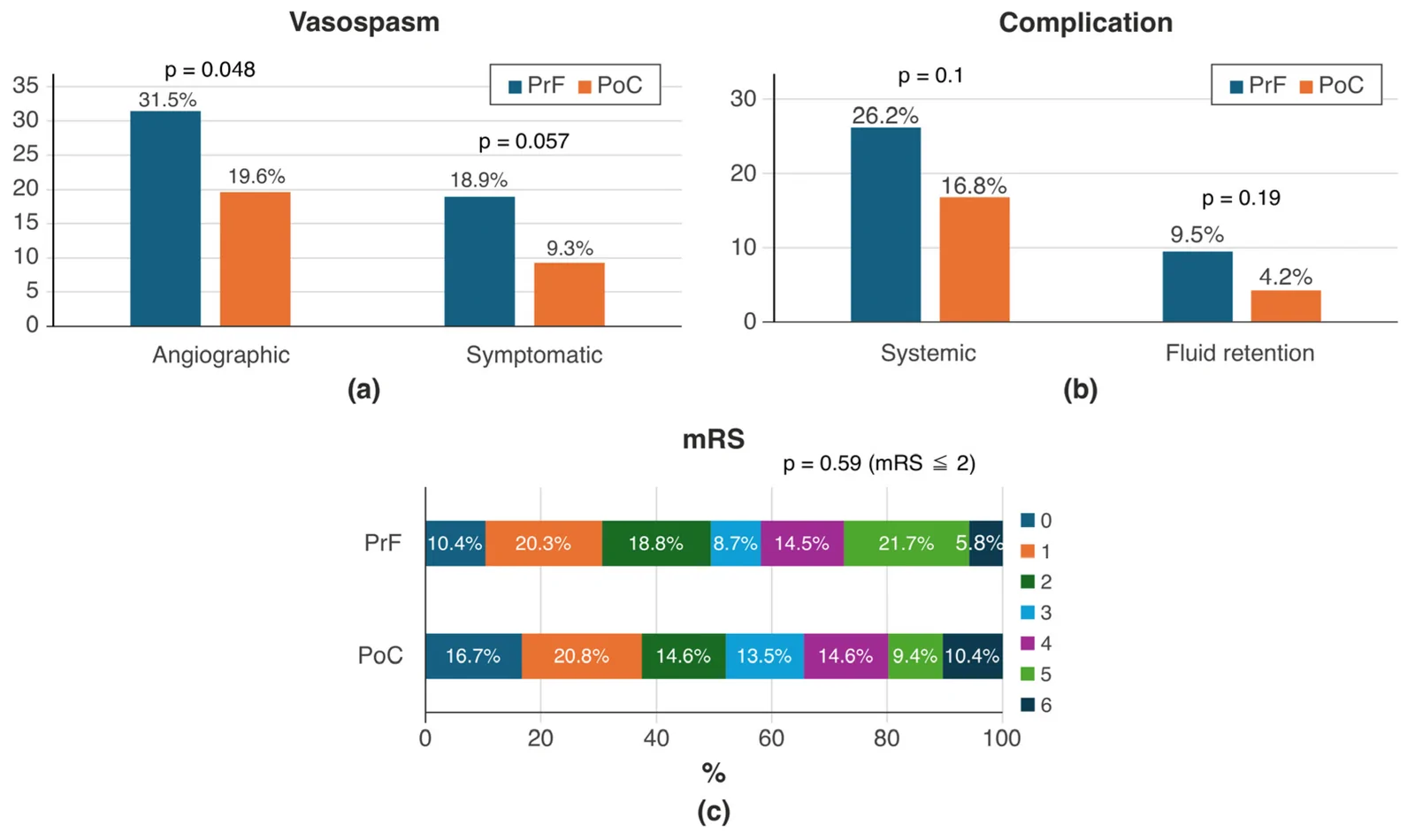
Comparison of outcomes between PrF and PoC. (**a**) Vasospasm; (**b**) complication; (**c**) mRS at discharge. PrF: preprotocol fasudil group, PoC: postprotocol clazosentan group, mRS modified Rankin Scale.

Patient characteristics and postoperative outcomes are summarized in Table 2 and Figure 2. There were no significant differences in patient backgrounds between the PrF and PoC groups. Regarding treatment, while the surgical methods employed showed no significant variation, the use of cilostazol and statins was notably lower in the PoC group (both *p* < 0.001). In terms of vasospasm occurrence, the PoC group exhibited significantly lower rates of angiographic vasospasm and had a lower incidence of symptomatic vasospasm compared to the PrF group (31.5% vs. 19.6%, *p* = 0.048; 18.9% vs. 9.3%, *p* = 0.057). However, no differences were observed between the groups concerning systemic complications, fluid retention, or the proportion of patients with an mRS score of 0–2. Logistic regression analysis, adjusted for variables including age, sex, WFNS grade, surgical method, and protocol, identified the clazosentan-based protocol as a significant predictive factor for reducing angiographic and symptomatic vasospasm (*p* = 0.02, adjusted odds ratio [OR] 0.48 [0.24–0.90]; *p* = 0.03, adjusted OR 0.4 [0.16–0.91]) (Table 3).

### 3.2. Comparison Between PrC and PoC

**Figure 3:**
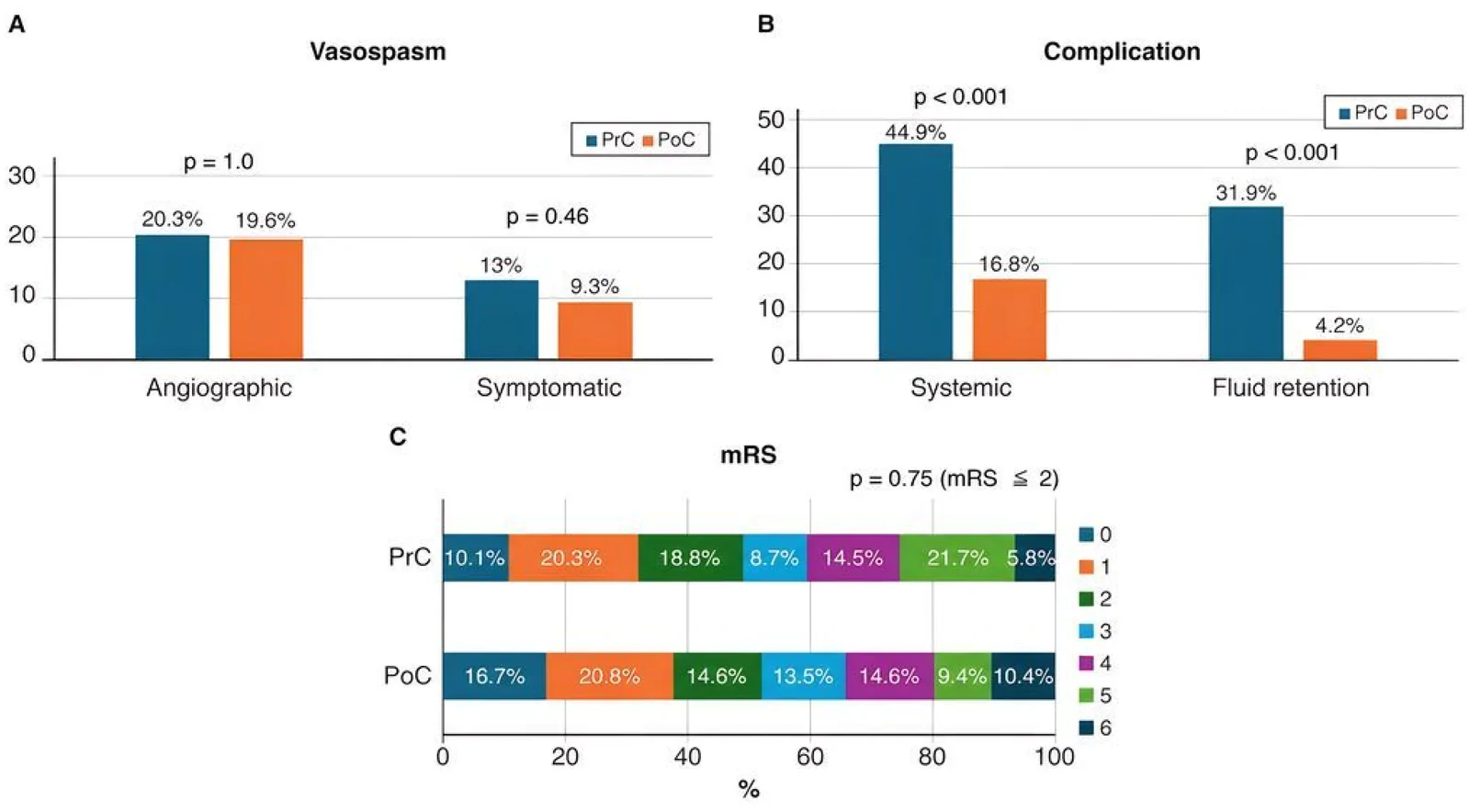
Comparison of outcomes between PrC and PoC. (**a**) Vasospasm; (**b**) complication; (**c**) mRS at discharge. PrC: preprotocol clazosentan group, PoC: postprotocol clazosentan group, mRS modified Rankin Scale.

No significant differences were noted in patient background characteristics between the PrC and PoC groups. Similarly, there were no significant differences in the occurrence of vasospasm, including radiological and symptomatic vasospasm. In terms of complications, however, the PoC group demonstrated significantly lower rates of systemic complications and fluid retention compared to the PrC group (44.9% vs. 16.8%, *p* < 0.001; 31.9% vs. 4.2%, *p* < 0.001) (Table 4 and Figure 3). No significant difference was observed between the two groups regarding mRS scores. Logistic regression analysis for systemic complications included interaction terms for protocol, age, sex, WFNS grade, and surgical method. The PoC group and younger age were identified as significant factors associated with a reduced incidence of systemic complications (*p* < 0.001, adjusted OR 0.24 [0.11–0.50]; *p* = 0.02, adjusted OR 1.03 [1.01–1.06]. For fluid retention, logistic regression revealed a significant reduction associated with direct surgery and protocol implementation (*p* = 0.009, adjusted OR 0.24 [0.06–0.71]; *p* < 0.001, adjusted OR 0.085 [0.02–0.25]) (Table 5).

### 3.3. Comparison Between PoF and PoC

A comparison was made between fasudil and clazosentan after the introduction of the protocol. The PoF group was older than the PoC group (74.5 ± 13.4 vs. 64 ± 14.6, *p* < 0.001), and the rate of endovascular treatment was higher in the PoF group compared to the PoC group (74.1% vs. 56.1%, *p* = 0.006). Regarding vasospasm, there were no significant differences in angiographic vasospasm between the two groups (28% vs. 19.6%, *p* = 0.41). However, symptomatic vasospasm tended to be lower in the PoC group (24% vs. 9.3%, *p* = 0.08). Logistic regression analysis showed that although the use of clazosentan was not statistically significant, a trend toward a reduction in the incidence of symptomatic vasospasm was observed (*p* = 0.076, OR 3.87 [0.868–17.258]). No significant differences were found between the two groups in terms of systemic complications, fluid retention, or the mRS at discharge (Table 6).

### 3.4. Comparison Between PrF and PoC in Elderly Patients (76 Years and Older)

A comparison was made between the PrF group and the PoC group (33 cases vs. 23 cases) for patients aged 76 and older. No significant differences were observed between the two groups in terms of angiographic vasospasm, symptomatic vasospasm, systemic complications, or fluid retention. Additionally, no differences were found in the mRS at discharge (Table 7). However, in the PoC group, a significant increase in fluid retention was noted when comparing patients aged 76 and older with those younger than 75 (13.4% vs. 1.4%, *p* = 0.042).

### 3.5. Comparison Between PrF and PoC in Patients with WFNS Grade V

A comparison was made between the PrF group and the PoC group (both 27 cases) for patients with WFNS grade V. There were no significant differences in the occurrence of vasospasm. Regarding systemic complications, the PoC group had significantly fewer cases compared to the PrF group (11.1% vs.40.7%, *p* = 0.028). Although no significant difference was observed, there was a trend toward reduced fluid retention in the PoC group compared to the PrF group (18.5% vs.0%, *p* = 0.051). No significant differences were found in the mRS between the two groups (Table 8).

## 4. Discussion

Clazosentan is a drug that selectively inhibits the binding of endothelin (ET) to the endothelin A receptor (ETA), preventing the increase in intracellular calcium levels in vascular smooth muscle cells, and thus inhibiting endothelin-induced cerebral vasospasm. Several studies have reported on the use of clazosentan for managing DCI after aSAH [1,2,3,4,5,6,7,8,9]. In this study, a multicenter collaborative investigation was conducted to evaluate treatment based on a standardized protocol, with clazosentan monotherapy used as the first-line treatment for cerebral vasospasm management.

### 4.1. PrF vs. PoC

The data in this study suggest that standardized protocols using clazosentan reduce the incidence of angiographic and symptomatic vasospasm without causing systemic complications or fluid retention. However, no improvement in the mRS at discharge was observed. Previous studies comparing clazosentan 5 mg/h with placebo for managing vasospasm after clipping found no significant differences in vasospasm or functional outcomes [6]. Additionally, comparisons of clazosentan 15 mg/h, clazosentan 5 mg/h, and placebo after coil embolization showed that while clazosentan 15 mg/h reduced vasospasm, none of the doses improved functional outcomes [7]. Even in trials using clazosentan at 15 mg/h, no significant differences in clinical outcomes were observed between the clazosentan and placebo groups [8]. However, both groups in these studies received nimodipine, which is not approved in Japan, complicating the evaluation of clazosentan’s effects alone. Additionally, it is important to consider the potential impact of different treatment initiation times. In contrast, Endo et al. reported in a phase Ⅲ trial that clazosentan 10 mg/h significantly reduced vasospasm, regardless of the surgical procedure, and improved the mRS [1]. While the dosing of clazosentan varies across these studies and results differ, it can be inferred that clazosentan at doses of 10 mg/h or higher may help prevent vasospasm. Reports comparing clazosentan with the traditionally used fasudil have shown that clazosentan reduces the incidence of symptomatic vasospasm [4,5]. The mRS results were obtained at acute care hospitals, and it is likely that patients were transferred for early rehabilitation. Kajiwara et al. [9] reported that although no significant difference was observed in mRS at discharge, the clazosentan group showed significant improvement compared to the ozagdrel sodium + fasudil hydrochloride group during follow-up 6 months later. It is believed that using clazosentan to prevent cerebral vasospasm, and consequently, secondary brain damage, may potentially improve mRS in the long term. Therefore, long-term follow-up is necessary to fully assess the treatment’s impact. Regarding cilostazol, its use was significantly reduced in the PoC group. A previous report [10] indicated that cilostazol is effective in preventing vasospasm and improving neurological outcomes. However, the results of this study suggest that it is plausible that a protocol based on clazosentan monotherapy could prevent vasospasm more effectively than the preprotocol treatment, without requiring cilostazol. This finding aligns with the study by Sakata et al. [5], which showed that clazosentan was associated with a significantly lower incidence of symptomatic vasospasm compared to fasudil combined with cilostazol. It is possible that clazosentan monotherapy could effectively reduce vasospasm, potentially simplifying treatment management.

### 4.2. PrC vs. PoC

Clazosentan side effects include fluid retention and pulmonary edema. One hypothesis suggests that selective inhibition of ETA reduces diuretic effects by activating arginine vasopressin and aldosterone through endothelin B (ETB) receptor stimulation [11]. It is also proposed that ETB stimulation promotes nitric oxide (NO) production, which in turn downregulates sodium–potassium ATPase (Na–K ATPase) activity in alveolar cells. This reduction in Na–K ATPase function impairs the reabsorption of interstitial fluid from the alveoli, leading to excessive fluid influx into the alveolar space and contributing to pulmonary edema [12]. Previous reports have indicated an increase in fluid retention, including pulmonary edema [5,7,13]. However, the data from this study suggest that a standardized protocol reduces the occurrence of systemic complications and fluid retention in patients treated with clazosentan. Specifically, closely monitoring for dehydration, restricting fluid intake to 1 mL/kg/h, and implementing regular imaging assessments as part of the protocol likely helped prevent these complications early on. Akamatsu et al. [13] reported that administering intravenous furosemide to manage fluid balance when it exceeds 1000 mL over 12 h could improve clazosentan therapy for vasospasm prevention. Herein, the objective of this study is to maintain the fluid balance at 0–500 mL. If the fluid balance exceeds this range, the infusion volume should be adjusted accordingly. Additionally, diuretics were administered when fluid balance exceeds 1500 mL, which, along with fluid intake restrictions, is believed to minimize complications, aid management during the vasospasm period, and ultimately ease the burden on patients and healthcare providers. Additionally, in cases where clazosentan was used, fluid retention decreased following direct surgery compared with that following interventional radiology. This decrease may have been influenced by factors such as the use of local anesthesia in endovascular surgery and difficulty in assessing intraoperative fluid balance, including the volume of catheter reflux fluid. Therefore, meticulous management of intraoperative fluid balance is essential across all treatment approaches, and to achieve standardized care, it is necessary to incorporate explicit guidelines for intraoperative fluid management into treatment protocols.

### 4.3. PoF vs. PoC

After the protocol was introduced, fasudil was more frequently used in elderly patients. This may be influenced by factors such as the exclusion of patients aged 76 and older in the phase Ⅲ trial of clazosentan [1] and the decreased cardiac function commonly seen in older individuals. Regarding vasospasm, clazosentan use tended to reduce symptomatic vasospasm, although the difference was not statistically significant. The lack of significant results may be due to the small sample size and variability in patient characteristics.

### 4.4. PrF vs. PoC in Elderly Patients (76 Years and Older)

The data from this study suggest that fasudil (PrF) and clazosentan (PoC) have similar performance in elderly patients, with no significant differences in safety outcomes, such as fluid retention and systemic complications, or other results when protocols are followed. This aligns with the findings of Mochizuki et al. [4], indicating that clazosentan can be safely used in elderly patients with similar efficacy to traditional fasudil-based treatment. However, in the PoC group, patients aged 76 and older were more prone to fluid retention compared to younger patients. It is considered that in elderly patients, more careful monitoring is needed than in younger patients. Mutoh et al. [14] reported that half of elderly patients treated with clazosentan developed pulmonary edema with hypoxemia, suggesting that older patients are more susceptible to fluid retention and face a higher risk of cardiopulmonary complications. Careful monitoring of urine volume, weight gain, and postoperative assessments is crucial in elderly patients, requiring more attention.

### 4.5. PrF vs. PoC in WFNS Grade V

Mochizuki et al. [4] reported that clazosentan significantly reduced DCI, but no significant difference in clinical outcomes between clazosentan and fasudil groups in patients with WFNS grade V was observed. In the current study, treatment with clazosentan according to the protocol showed no significant difference in vasospasm compared to fasudil treatment. However, systemic complications were significantly reduced, and while not statistically significant, there was a trend toward reduced fluid retention. Reducing complications may aid in postoperative management. However, there was no significant difference in mRS at discharge between the two groups, suggesting that while the PoC group had better management outcomes, the severity of WFNS grade V may limit overall outcomes.

### 4.6. Limitation

This study has several limitations. First, although treatment followed a standardized protocol, the final decision was made by each institution and attending neurosurgeon based on the patient’s condition, which may introduce bias. Second, diagnostic assessments for vasospasm, systemic complications, and fluid retention were conducted by the attending physicians, leading to a lack of full standardization. Third, the protocol does not address fluid balance during the preoperative and intraoperative periods, focusing only on the postoperative phase. Fourthly, due to differences in the timing of clazosentan introduction at various institutions, a transition period occurred in protocol implementation, resulting in a complex group classification. Finally, due to the lack of long-term follow-up data, the definitive benefit of protocol-based treatment on long-term outcomes remains unclear and is an important issue to be addressed in future studies.

## 5. Conclusions

Treatment following a protocol that prioritizes clazosentan monotherapy as the first-line option may reduce the occurrence of vasospasm compared to conventional treatment, with similar safety profiles, while also decreasing the incidence of fluid retention, a known side effect of clazosentan. This approach may simplify postoperative management and alleviate the burden on patients as well as healthcare providers. Furthermore, although additional studies with larger sample sizes are needed, clazosentan has shown potential for safe use in elderly patients and those with WFNS grade V, with efficacy comparable to conventional treatment.

## Figures and Tables

**Table 1:** Standardized protocol overview.

Initial response	Stabilize respiration and circulation (consider endotracheal intubation) Ensure adequate sedation and analgesia Lower blood pressure (sBP 100–140 mmHg)
Initial examination	Diagnosis of SAH with CT, MRI (FLAIR). Detection of aneurysm with CTA, or angiography if necessary Contrast-enhanced MRI to identify the rupture site
Preoperative management	Avoid invasive procedures (e.g., urethral balloon, gastric tube after sedation) Maintain blood pressure (sBP 100–140 mmHg) Head elevated by 20° Cardiology evaluation for pulmonary edema, takotsubo cardiomyopathy, etc.
Surgical intervention	Direct or endovascular surgery (based on facility and patient)
Postoperative management	Postoperative BP: sBP 120–160 mmHg **Prevention of DCI:** First choice: Clazosentan (10 mg/h) Others: Fasudil (60 mg/day) or Ozagrel sodium (80 mg/day) The use of cilostazol and statins is facility-specific **Fluid management:** Normovolemia Basic infusion: extracellular fluid (1 mL/kg/h) Target water balance (0–500 mL); infusion volume was adjusted in case of overbalance. Diuretics: in case of fluid overload (over 1500 mL) Monitor for CSWS and SIADH (Na 130–150 mEq/L) Hb > 8 g/dL, Alb > 2.5 g/dL, Blood glucose < 180 mg/dL, Normothermia Administering antiulcer medications, prefer enteral feeding for oral intake difficulties
CSF management	Manage SD, VD, and CD (drainage volume: 150–250 mL/day)
Postoperative examination	CT (day after surgery and around day 4), CTA (1 week after clipping), chest CT (at the same time as head CT, if possible) MRI and MRA (day after surgery, around day 8, day 14, and any time of suspected vasospasm) Chest X-ray (as needed) Blood tests (every other day)
Treatment for DCI	Extracellular fluid loading Increase target blood pressure Consider endovascular treatment
Rehabilitation	Implemented to prevent contractures and maintain ADL

sBP, systolic blood pressure, CT, computed tomography; MRI, magnetic resonance imaging; FLAIR, fast fluid attenuated inversion recovery; CTA, CT angiography; DCI, delayed cerebral ischemia; CSWS, central salt wasting syndrome; SIADH, syndrome of inappropriate antidiuretic hormone secretion; Hb, hemoglobin; Alb, albumin; SD, spinal drainage; VD, ventricle drainage; CD, cisternal drainage; ADL, activity of daily living.

**Table 2:** Comparison of patients’ characteristics between PrF and PoC.

	PrF (*n* = 128)	PoC (*n* = 97)	*p*-Value
**Age (year)**	65 ± 14.2	64 ± 14.6	0.78
**Sex (female)**	101 (78.9%)	68 (70.1%)	0.16
**Past history**			
**Hypertension**	56 (43.8%)	39 (40.2%)	0.68
**Diabetes mellitus**	10 (7.8%)	4 (4.1%)	0.4
**Past stroke**	11 (8.6%)	5 (5.2%)	0.43
**WFNS grade**			0.2
**I**	39 (30.5%)	21 (21.7%)	
**II**	29 (22.7%)	24 (24.7%)	
**III**	12 (9.4%)	15 (15.5%)	
**IV**	21 (16.4%)	10 (10.3%)	
**V**	27 (21.1%)	27 (27.8%)	
**Fisher group**			0.12
**1**	6 (4.7%)	2 (2.1%)	
**2**	10 (7.8%)	6 (6.2%)	
**3**	102 (79.7%)	72 (74.2%)	
**4**	10 (7.8%)	17 (17.5%)	
**Treatment**			0.07
**IVR**	82 (65.6%)	50 (51.6%)	
**DS**	43 (34.4%)	46 (47.4%)	
**Combined**	0 (0)	1 (1%)	
**Spinal drainage**	75 (59.5%)	61 (63.5%)	0.58
**Cilostazol**	94 (74.0%)	50 (51.6%)	<0.001
**Statin**	88 (68.8%)	39 (40.2%)	<0.001

PrF: preprotocol fasudil group, PoC: postprotocol clazosentan group, WFNS: World Federation of Neurosurgical Societies, IVR: interventional radiology, DS: direct surgery.

**Table 3:** Univariate and multivariate analysis for factors associated with vasospasm.

		Vasospasm (Yes) (*n* = 59/33)	Vasospasm (No) (*n* = 166/191)	Univariate Analysis *p*-Value	Multivariate Analysis *p*-Value (Adjusted OR [95% CI])
**Angiographic vasospasm**	**Age**	66.0 ± 13.6	63.9 ± 14.5	0.33	0.21 (1.01 [0.99–1.04])
	**Sex (female)**	39 (66.1%)	129(78.2%)	0.08	0.025 (0.45 [0.22–0.90])
	**WFNS grade** **IV** **,** **V**	28 (47.4%)	56 (33.9%)	0.08	0.07 (1.8 [0.96–3.40])
	**Operation** **DS**	22 (37.3%)	67 (41.4%)	0.64	0.94 (1.8 [0.51–1.86])
	**Postprotocol**	19 (32.2%)	78 (47.3%)	0.048	0.02 (0.48 [0.24–0.90])
**Symptomatic vasospasm**	**Age**	65.4 ± 12.8	64.3 ± 14.5	0.67	0.51 (1.97 [0.23–17.6])
	**Sex (female)**	21 (63.6%)	147 (77%)	0.13	0.053 (0.43 [0.19–1.01])
	**WFNS grade** **IV** **,** **V**	16 (48.5%)	67 (25.6%)	0.18	0.17 (1.73 [0.79–3.78])
	**Operation** **DS**	11 (33.3%)	78 (41.5%)	0.44	0.64 (0.82 [0.35–1.84])
	**Postprotocol**	9 (27.3%)	103 (53.9%)	0.057	0.03 (0.4 [0.16–0.91])

OR: Odds ratio, CI: Confidence Interval, WFNS: World Federation of Neurosurgical Societies, DS: direct surgery, IVR: interventional radiology.

**Table 4:** Comparison of patient characteristics and outcomes between PrC and PoC.

	PrC (*n* = 69)	PoC (*n* = 97)	*p*-Value
**Age (year)**	69 ± 16.3	64 ± 14.6	0.50
**Sex (female)**	45 (65.2%)	68 (70.1%)	0.61
**Past history**			
**Hypertension**	35 (50.7%)	39 (40.2%)	0.21
**Diabetes mellitus**	3 (4.4%)	4 (4.1%)	1.0
**Past stroke**	7 (10.1%)	5 (5.2%)	0.24
**WFNS grade**			0.1
**I**	17 (25%)	21 (21.7%)	
**II**	15 (22.1%)	24 (24.7%)	
**III**	3 (4.4%)	15 (15.5%)	
**IV**	14 (20.6%)	10 (10.3%)	
**V**	19 (27.9%)	27 (27.8%)	
**Fisher group**			0.22
**1**	1 (1.5%)	2 (2.1%)	
**2**	5 (7.3%)	6 (6.2%)	
**3**	42 (60.9%)	72 (74.2%)	
**4**	21 (30.4%)	17 (17.5%)	
**Treatment**			0.09
**IVR**	44 (66.7%)	50 (51.6%)	
**DS**	22 (33.3%)	46 (47.4%)	
**Combined**	0 (0%)	1 (1%)	
**Spinal drainage**	41 (60.3%)	61 (63.5%)	0.74
**Cilostazol**	29 (42%)	50 (51.6%)	0.27
**Statin**	31 (44.9%)	39 (40.2%)	0.63

PrC: preprotocol clazosentan group, PoC: postprotocol clazosentan group, WFNS: World Federation of Neurosurgical Societies, IVR: interventional radiology, DS: direct surgery.

**Table 5:** Univariate and multivariate analyses of factors associated with complications.

		Complication (Yes) (*n* = 47/26)	Complication (No) (*n* = 117/139)	Univariate Analysis *p*-Value	Multivariate Analysis *p*-Value (Adjusted OR [95% CI])
**Systemic complication**	**Age**	68.2 ± 15.1	62.9 ± 15.0	0.046	0.02 (1.03 [1.01–1.06])
	**Sex (female)**	31 (66.0%)	81 (72.3%)	0.71	0.22 (0.57 [0.23–1.4])
	**WFNS grade** **IV** **,** **V**	19 (40.4%)	51 (44%)	0.73	0.2 (0.6 [0.27–1.3])
	**Operation** **DS**	13 (28.3%)	53 (46.1%)	0.05	0.09 (0.5 [0.22–1.1])
	**Postprotocol**	16 (34.0%)	79 (67.5%)	<0.001	<0.001 (0.24 [0.11–0.50])
**Fluid** **retention**	**Age**	68.6 ± 15.3	63.8 ± 15.2	0.14	0.15 (1.03 [0.99–1.07])
	**Sex (female)**	19 (73.1%)	94 (67.6%)	0.65	0.87 (0.91 [0.27–3.2])
	**WFNS grade** **IV** **,** **V**	11 (42.3%)	59 (42.8%)	1.0	0.39 (0.65 [0.24–1.7])
	**Operation** **DS**	4 (15.4%)	63 (46.3%)	0.004	0.009 (0.24 [0.06–0.71])
	**Postprotocol**	4 (15.4%)	92 (66.2%)	<0.001	<0.001 (0.085 [0.02–0.25])

OR: Odds ratio, CI: confidence interval, WFNS: World Federation of Neurosurgical Societies, DS: direct surgery, IVR: interventional radiology.

**Table 6:** Comparison of patient characteristics and outcomes between PoF and PoC.

	PoF (*n* = 28)	PoC (*n* = 97)	*p*-Value
**Age (year)**	74.5 ± 13.4	64 ± 14.6	<0.001
**Sex (female)**	21 (75%)	68 (70.1%)	0.81
**Past history**			
**Hypertension**	15 (53.6%)	39 (40.2%)	0.28
**Diabetes mellitus**	1 (3.6%)	4 (4.1%)	1.0
**Past stroke**	3 (10.7%)	5 (5.2%)	0.38
**WFNS grade**			0.54
**I**	7 (25%)	21 (21.7%)	
**II**	9 (32.1%)	24 (24.7%)	
**III**	1 (3.6%)	15 (15.5%)	
**IV**	3 (10.7%)	10 (10.3%)	
**V**	8 (28.6%)	27 (27.8%)	
**Fisher group**			0.3
**1**	2 (2.1%)	2 (2.1%)	
**2**	2 (2.1%)	6 (6.2%)	
**3**	17 (60.7%)	72 (74.2%)	
**4**	7 (25%)	17 (17.5%)	
**Treatment**			0.006
**IVR**	20 (74.1%)	50 (51.6%)	
**DS**	5 (18.5%)	46 (47.4%)	
**Combined**	2 (7.4%)	1 (1%)	
**Spinal drainage**	16 (57.1%)	61 (63.5%)	0.66
**Cilostazol**	16 (57.1%)	50 (51.6%)	0.67
**Statin**	16 (57.1%)	39 (40.2%)	0.13
**Vasospasm**			
**Angiographic**	7 (28.0%)	19 (19.6%)	0.41
**Symptomatic**	6 (24.0%)	9 (9.3%)	0.08
**Complication**			
**Systemic**	4 (14.3%)	16 (16.8%)	1.0
**Fluid retention**	1 (3.6%)	4 (4.2%)	1.0
**mRS 0–2 at discharge**	11 (39.3%)	50 (52.1%)	0.29

PoF: postprotocol fasudil group, PoC: postprotocol clazosentan group, WFNS: World Federation of Neurosurgical Societies, IVR: interventional radiology, DS: direct surgery, mRS: modified Rankin Scale.

**Table 7:** Comparison between PrF and PoC in elderly patients.

Elderly (>75)	PrF (*n* = 33)	PoC (*n* = 23)	*p*-Value
**Age (year)**	83.3 ± 5.7	82.3 ± 5.3	0.56
**Sex (female)**	32 (97.0%)	20 (87.0%)	0.29
**Past history**			
**Hypertension**	18 (54.6%)	14 (60.9%)	0.78
**Diabetes mellitus**	3 (9.1%)	0 (0%)	0.26
**Past stroke**	3 (9.1%)	2 (8.7%)	1.0
**WFNS grade**			0.41
**I**	8 (24.2%)	6 (26.1%)	
**II**	3 (9.1%)	4 (17.4%)	
**III**	3 (9.1%)	4 (17.4%)	
**IV**	9 (27.3%)	2 (8.7%)	
**V**	10 (30.3%)	7 (30.4%)	
**Fisher group**			0.86
**1**	1 (3.0%)	0 (0%)	
**2**	1 (3.0%)	2 (8.7%)	
**3**	27 (81.8%)	18 (78.3%)	
**4**	4 (12.1%)	3 (13.0%)	
**Treatment**			0.55
**IVR**	22 (68.8%)	14 (60.9%)	
**DS**	10 (31.3%)	8 (34.8%)	
**Combined**	0 (0%)	1 (4.4%)	
**Spinal drainage**	20 (62.5%)	17 (73.9%)	0.4
**Cilostazol**	29 (87.9%)	10 (43.5%)	<0.001
**Statin**	20 (60.6%)	7 (30.4%)	0.03
**Vasospasm**			
**Angiographic**	13 (40.6%)	5 (21.7%)	0.16
**Symptomatic**	8 (25.0%)	2 (8.7%)	0.17
**Complication**			
**Systemic**	13 (40.6%)	5 (22.7%)	0.24
**Fluid retention**	3 (9.4%)	3 (13.0%)	0.69
**mRS 0–2 at discharge**	6 (20.7%)	5 (21.7%)	1.0

PrF: preprotocol fasudil group, PoC: postprotocol clazosentan group, WFNS: World Federation of Neurosurgical Societies, IVR: interventional radiology, DS: direct surgery, mRS: modified Rankin Scale.

**Table 8:** Comparison between PrF and PoC in patients with WFNS grade V.

WFNS Grade V	PrF (*n* = 27)	PoC (*n* = 27)	*p*-Value
**Age (year)**	66.9 ± 15.4	66.4 ± 13.2	0.94
**Sex (female)**	21 (77.8%)	17 (63.0%)	0.37
**Past history**			
**Hypertension**	16 (59.3%)	9 (33.3%)	0.10
**Diabetes mellitus**	2 (7.4%)	1 (3.7%)	1.0
**Past stroke**	2 (7.4%)	2 (7.4%)	1.0
**Fisher group**			0.25
**1**	0 (0%)	0 (0%)	
**2**	0 (0%)	0 (0%)	
**3**	20 (74.1%)	15 (55.6%)	
**4**	7 (25.9%)	12 (44.4%)	
**Treatment**			0.03
**IVR**	21 (80.8%)	13 (48.2%)	
**DS**	5 (19.2%)	13 (48.2%)	
**Combined**	0 (0%)	1 (3.7%)	
**Spinal drainage**	17 (63%)	15 (57.7%)	0.78
**Cilostazol**	20 (74.1%)	11 (40.7%)	0.027
**Statin**	17 (63.0%)	12 (44.4%)	0.27
**Vasospasm**			
**Angiographic**	10 (37.0%)	7 (25.9%)	0.56
**Symptomatic**	6 (22.2%)	3 (11.1%)	0.47
**Complication**			
**Systemic**	11 (40.7%)	3 (11.1%)	0.028
**Fluid retention**	5 (18.5%)	0 (0%)	0.051
**mRS 0–2 at discharge**	7 (29.2%)	8 (30.8%)	1.0

PrF: preprotocol fasudil group, PoC: postprotocol clazosentan group, WFNS: World Federation of Neurosurgical Societies, IVR: interventional radiology, DS: direct surgery, mRS: modified Rankin Scale.

## Data Availability

The raw data supporting the conclusions of this article will be made available by the authors on request.

## Abbreviations

The following abbreviations are used in this manuscript:

aSAH	aneurysmal subarachnoid hemorrhage
DCI	delayed cerebral ischemia
ET	endothelin
ETA	endothelin A receptor
ETB	endothelin B receptor
mRS	modified Rankin Scale
Na–K ATPase	sodium–potassium ATPase
PoC	postprotocol clazosentan
PoF	postprotocol fasudil
PrC	preprotocol clazosentan
PrF	preprotocol fasudil
WFNS	World Federation of Neurosurgical Societies
