# Correction: Kondo et al. Multicenter Validation of a Unified Evidence-Based Treatment Protocol Focusing on Clazosentan for Managing Subarachnoid Hemorrhage. *J. Clin. Med.* 2025, *14*, 3423

**DOI:** 10.3390/jcm15208032

**Published:** 2026-10-16

**Authors:** Hiroshi Kondo, Daizo Ishii, Masashi Kuwabara, Takeshi Hara, Kaoru Kurisu, Masayuki Sumida, Fusao Ikawa, Shinji Ohba, Atsushi Tominaga, Naohiko Obayashi, Kazuhiko Kuroki, Takashi Sadatomo, Osamu Hamasaki, Shigeyuki Sakamoto, Toshinori Matsushige, Yosuke Watanabe, Hayato Araki, Masaru Abiko, Nobuhiko Ichinose, Atsumi Takenobu, Nobutaka Horie

**Affiliations:** 1 Department of Neurosurgery, Graduate School of Biomedical and Health Sciences, Hiroshima University, Hiroshima 734-8551, Japan; daiishii@hiroshima-u.ac.jp (D.I.); m214028@gmail.com (M.K.); thara@hiroshima-u.ac.jp (T.H.); horie@hiroshima-u.ac.jp (N.H.); 2 Department of Neurosurgery, National Hospital Organization Kure Medical Center and Chugoku Cancer Center, Kure 737-0023, Japan; s-ooba@seifu.net; 3 Chugoku Rosai Hospital, Kure 737-0193, Japan; kuka422@chugokuh.johas.go.jp; 4 Department of Neurosurgery, Hiroshima Red Cross Hospital and Atomic-bomb Survivors Hospital, Hiroshima 730-8619, Japan; sumidamasa@gmail.com; 5 Department of Neurosurgery, Shimane Prefectural Central Hospital, Izumo 693-8555, Japan; fikawa@hiroshima-u.ac.jp; 6 Department of Neurosurgery and Neuroendovascular Therapy, Hiroshima Prefectural Hospital, Hiroshima 734-8530, Japan; atomin1963@gmail.com; 7 Department of Neurosurgery, Matsue Red Cross Hospital, Matsue 690-8506, Japan; n972061@yahoo.co.jp; 8 Department of Neurosurgery, JA Hiroshima General Hospital, Hatsukaichi 738-8503, Japan; kkurocky6576@yahoo.co.jp; 9 Department of Neurosurgery, National Hospital Organization Higashihiroshima Medical Center, Higashihiroshima 739-0041, Japan; tasanetomo@gmail.com; 10 Department of Neurosurgery, Miyoshi Central Hospital, Miyoshi 728-8502, Japan; o.hamasaki7745@city.miyoshi.hiroshima.jp; 11 Department of Neurosurgery, Itsukaichi Memorial Hospital, Itsukaichi 731-5156, Japan; shigeyukisakamoto@gmail.com; 12 Department of Neurosurgery and Interventional Neuroradiology, Hiroshima City North Medical Center Asa Citizens Hospital, Hiroshima 731-0293, Japan; teruteru728@yahoo.co.jp; 13 Department of Neurosurgery, Matsuyama Red Cross Hospital, Matsuyama 790-8524, Japan; watanabeyosuke2001@yahoo.co.jp; 14 Department of Neurosurgery, Araki Neurosurgical Hospital, Hiroshima 733-0821, Japan; haraki@arakihp.jp; 15 Department of Neurosurgery, JA Onomichi General Hospital, Onomichi 722-8508, Japan; tenma578@gmail.com; 16 Department of Neurosurgery, Ichinose Hospital, Hiroshima 730-0042, Japan; n.1s.stream@gmail.com; 17 Department of Neurosurgery, Teraoka Memorial Hospital, Fukuyama 729-3103, Japan; neurostake@gmail.com

**Error in Figure 3**

In the original publication [1], there was a mistake in Figure 3C as published. Regarding the modified Rankin Scale (mRS) at discharge for the PrC group, the value was incorrectly shown as 29.3%, whereas the correct value is 20.3%. This error occurred during figure preparation because of an incorrect numerical entry in Excel.

This error is limited to the figure only. The underlying dataset, statistical analyses, results, and conclusions of the manuscript remain unchanged. The authors state that the scientific conclusions are unaffected. This correction was approved by the Academic Editor. The original publication has also been updated.

The corrected Figure 3 appears below.


